# Evolution of the heteroharmonic strategy for target-range computation in the echolocation of Mormoopidae

**DOI:** 10.3389/fphys.2013.00141

**Published:** 2013-06-12

**Authors:** Emanuel C. Mora, Silvio Macías, Julio Hechavarría, Marianne Vater, Manfred Kössl

**Affiliations:** ^1^Research Group of Bioacoustics and Neuroethology, Department of Animal and Human Biology, Faculty of Biology, Havana UniversityHavana, Cuba; ^2^Institut für Zellbiologie und Neurowissenschaft, J.W. Goethe Universität FrankfurtFrankfurt am Main, Germany; ^3^Institut Biochemie und Biologie, Allgemeine Zoologie, Universität PotsdamPotsdam, Germany

**Keywords:** target-range, echolocation, heteroharmonic computation, Mormoopidae, call-echo delay

## Abstract

Echolocating bats use the time elapsed from biosonar pulse emission to the arrival of echo (defined as echo-delay) to assess target-distance. Target-distance is represented in the brain by delay-tuned neurons that are classified as either “heteroharmonic” or “homoharmormic.” Heteroharmonic neurons respond more strongly to pulse-echo pairs in which the timing of the pulse is given by the fundamental biosonar harmonic while the timing of echoes is provided by one (or several) of the higher order harmonics. On the other hand, homoharmonic neurons are tuned to the echo delay between similar harmonics in the emitted pulse and echo. It is generally accepted that heteroharmonic computations are advantageous over homoharmonic computations; i.e., heteroharmonic neurons receive information from call and echo in different frequency-bands which helps to avoid jamming between pulse and echo signals. Heteroharmonic neurons have been found in two species of the family Mormoopidae (*Pteronotus parnellii* and *Pteronotus quadridens*) and in *Rhinolophus rouxi*. Recently, it was proposed that heteroharmonic target-range computations are a primitive feature of the genus *Pteronotus* that was preserved in the evolution of the genus. Here, we review recent findings on the evolution of echolocation in Mormoopidae, and try to link those findings to the evolution of the heteroharmonic computation strategy (HtHCS). We stress the hypothesis that the ability to perform heteroharmonic computations evolved separately from the ability of using long constant-frequency echolocation calls, high duty cycle echolocation, and Doppler Shift Compensation. Also, we present the idea that heteroharmonic computations might have been of advantage for categorizing prey size, hunting eared insects, and living in large conspecific colonies. We make five testable predictions that might help future investigations to clarify the evolution of the heteroharmonic echolocation in Mormoopidae and other families.

## Introduction

Echolocation allows bats to create perceptual images of complex night environments (Griffin, 1959; Moss and Surlykke, 2010). A key piece of information obtained during echolocation is the space-depth of surrounding objects that constitute possible targets (Simmons, 1973, 2012; Wenstrup and Suthers, 1984). Target distance is assessed from the time delay between the outgoing call and the returning echo (Simmons, 1971; Simmons et al., 1979). The central auditory system of echolocating bats contains specialized neurons that respond to particular call-echo delays (Feng et al., 1978; O'Neill and Suga, 1979). The combined activity of populations of delay-tuned neurons presumably determines the bat's ability for target-range computation (Suga, 1990; Simmons, 2012).

The neural processing of target-distance has been studied in six bat species from four different families: Mormoopidae (*Pteronotus parnellii*; O'Neill and Suga, 1979; Suga et al., 1979 and *Pteronotus quadridens*, Hechavarría et al., 2013); Rhinolophidae (*Rhinolophus rouxi*; Schuller et al., 1991); Vespertilionidae (*Myotis lucifugus*; Sullivan, 1982; Wong and Shannon, 1988 and *Eptesicus fuscus*; Feng et al., 1978; Dear et al., 1993); and Phyllostomidae (*Carollia perspicillata*; Hagemann et al., 2010, 2011). Two different neuronal strategies for target-range computation have been identified. In bats that broadcast frequency-modulated (FM) calls, delay-tuned neurons respond to similar harmonics in the calls and echoes, thus employing a homoharmonic computation strategy (HmHCS) (Feng et al., 1978; Sullivan, 1982; Dear et al., 1993; Hagemann et al., 2010). In two bat species from the family Mormoopidae (*P. parnellii* and *P. quadridens*) and one species from the family Rhinolophidae (*R. rouxi*) delay-tuned neurons are activated by the combination of the FM component of the fundamental harmonic in the call and one of the higher harmonic FM components in the echo (O'Neill and Suga, 1979; Suga et al., 1979; Schuller et al., 1991).

The “heteroharmonic computation strategy” (HtHCS) was first described in *P. parnellii* (Suga et al., 1978) and *R. rouxi* (Schuller et al., 1991). These two bat species use echolocation calls that combine long constant-frequency (CF) and FM components. For this reason it was long believed that HtHCS was an exclusive feature of the so called long CF-bats (Schuller et al., 1991; Wenstrup and Portfors, 2011). Recently, Hechavarría et al. (2013) reported that HtHCS is also a feature of neurons in the auditory cortex of the mormoopid *P. quadridens*, a species that uses short CF (sCF)-FM echolocation (Macías and Mora, 2003; Macías et al., 2006). That *P. quadridens* is able to use HtHCS echolocation is interesting not only from a functional point of view but also from an evolutionary angle, since (to our knowledge) Mormoopidae is the only family of bats including both CF-FM and sCF-FM species.

The evolution of echolocation has received much attention in the last decade. Recent molecular phylogenies (Eick et al., 2005; Teeling et al., 2005) have shaped new perspectives on the evolution of bat echolocation behavior (Jones and Teeling, 2006). Signal design (Jones and Holderied, 2007), duty cycle (Fenton et al., 2012), call frequency (Stoffberg et al., 2011), and Doppler shift compensation (Schnitzler and Denzinger, 2011) have been reviewed in the light of new phylogenetic insights.

In this review, we explore the evolution of the HtHCS in bat species from the family Mormoopidae. There are several recent findings that motivated this work. (1) HtHCS was found in *P. quadridens* (Hechavarría et al., 2013). (2) The CF-bat *P. parnellii* holds a basal position in the lineage of the genus *Pteronotus* (Van den Bussche and Weyandt, 2003; Dávalos, 2006). (3) The auditory cortex of newborn bats that do not yet echolocate is equipped with a set of fully functional delay-tuned neurons (Kössl et al., 2012) which suggests that target-range computation strategies could be genetically pre-determined. (4) A scheme for the evolution of “Doppler shift compensation” by bats of the family Mormoopidae was proposed (Smotherman and Guillen-Servent, 2008). (5) New call designs, activity patterns and diets were described in Caribbean mormoopids (Mora et al., 2011; Goerlitz et al., 2012; Mancina et al., 2012; Rolfe and Kurta, 2012).

We discuss how brain adaptations, distinctive characteristics of calls- and echoes- and phylogenetic relationships in mormoopids could have led to the acquisition of the heteroharmonic target-range computation strategy in this family. We argue that the HtHCS provides mormoopids with behavioral and ecological advantages for categorizing prey-size, hunting eared insects, and living in large colonies. By conducting the analysis in the light of recent molecular phylogenies, we are able to explore the evolutionary relationships between HtHCS and CF-specializations. We present the hypothesis that in Mormoopidae, HtHCS echolocation evolved independently from long-CF echolocation, high duty cycle (HDC) echolocation and Doppler Shift Compensation. We make five specific, testable predictions that might help future investigations to decipher the evolution of the heteroharmonic echolocation in Mormoopidae and other families.

## Delay tuning in auditory neurons of different bat species

**Figure 1:**
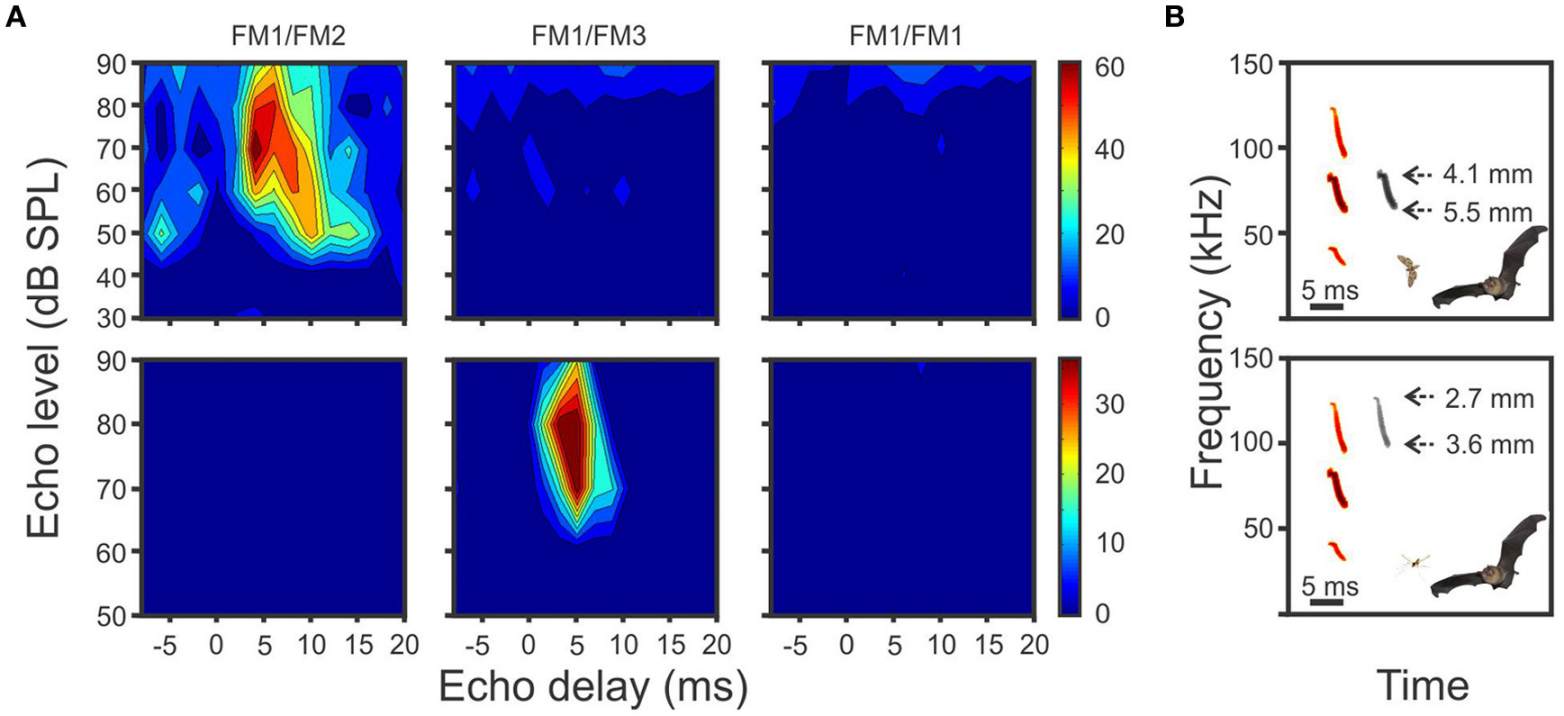
**(A)** Example delay response areas (DRAs) of two units from *P. quadridens*. The units were tuned to different harmonic combinations. Responses of the same unit were aligned horizontally. **(B)** The range of theoretical insect sizes generated from echoes of each biosonar harmonic. Insect size was calculated according to the maximum and minimum frequencies in each harmonic after Macías et al. (2006). It is suggested that each neuron could play a role in prey categorization according to size.

The most commonly used approach to determine whether a neuron is tuned to echo-delay or not consists in presenting the animal with artificial (or natural) pulse-echo pairs with different delays. The response of the neurons is measured as the number of spikes fired by the neuron in response to each echo-delay. If the echo-level is also changed during the recording, then the neuronal response is represented in the two dimensional space of echo-delay and echo-level in the form of a delay response area (DRA). Delay tuned neurons respond only (or maximally) to a few combinations of echo-delay and echo-level (see examples DRAs in Figure 1).

Different methods have been used to study the harmonic sensitivity of delay-tuned neurons in different bat species. By deleting components of the echolocation call and echo, Suga and co-workers (Suga et al., 1983) demonstrated that in the cortex of *P. parnellii*, the maximum response of delay-tuned neurons occurs when the fundamental FM-harmonic in the biosonar pulse (FM1) is followed by one of the upper FM-harmonics in the echo (i.e., FM2, FM3, or FM4) with a certain delay. Delay-tuned neurons are classified according to their best harmonic combination, i.e., the combination of pulse and echo harmonic that elicits the largest response. In *P. parnellii*, neurons tuned to combinations of FM1 and FM2, FM1-FM3, and FM1-FM4 have been found (Suga et al., 1983; Hagemann et al., 2011). Heteroharmonic neurons can be found in newborn *P. parnellii* long before they start to echolocate (Kössl et al., 2012). The latter could indicate that this neuronal ability is imprinted in the genome of the species and therefore it could have been subjected to evolutionary pressures.

In *P. quadridens* the frequency profile of cortical delay-tuned neurons was studied by presenting the bat with combinations of different harmonic components that included FM1/FM2, FM1/FM3, FM1/FM1, and FM2/FM2 (Hechavarría et al., 2013). The frequency profile of delay-tuned neurons in *P. quadridens* is quite similar to the frequency profile of delay-tuned neurons of *P. parnellii*. Example heteroharmonic neurons of *P. quadridens* are shown in Figure 1A. The delay-tuned neurons of *P. quadridens* fire only (or more strongly) in response to heteroharmonic pulse echo-pair combinations, i.e., FM1/FM2 and FM1/FM3. It has been suggested that neurons tuned to different harmonic combinations could provide information about targets with different acoustic properties i.e., preys of different sizes (Figure 1B).

**Figure 2:**
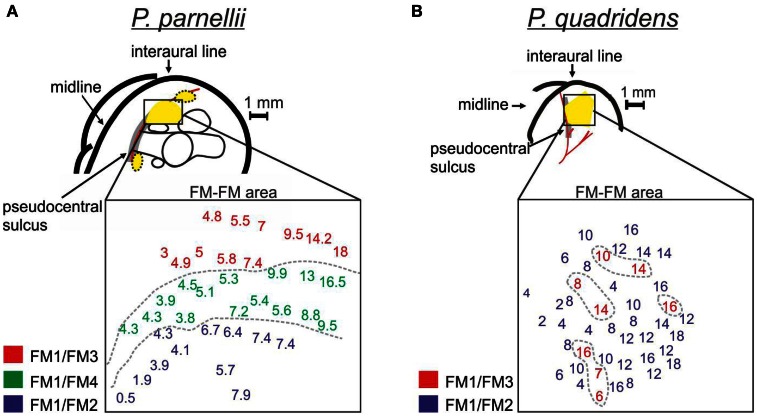
**Topographic organization of the FM/FM area of (A) *P. parnellii* and (B) *P. quadridens*.** In each species, schematic representations of the brain are given. In the schematic brain representations, prominent landmarks and blood vessels are indicated. The yellow areas indicate cortical regions dominated by delay-tuned neurons. Note that in the dorsal auditory cortex of both species, close to the pseudocentral sulcus, there are large areas dedicated to the processing of call-echo delay. In each species, a detailed map of the FM/FM area is given. The data from *P. parnellii* is from one specimen [modified from Hagemann et al. (2011)]. The data from *P. quadridens* was pooled from 3 specimens [see Hechavarría et al. (2013) for methods for reconstruction of cortical maps]. In FM/FM area maps, numbers positioned at the coordinates of each neuron indicate characteristic delays. Numbers were color-coded to indicate the best harmonic combination of each neuron. Note that in *P. parnellii* neurons processing different harmonic combinations form different clusters in the cortical surface. However, in *P. quadridens*, neurons processing FM1/FM2 and FM1/FM3 are intermixed. In both species neurons processing shorter delays are located rostrally and those processing longer delays are located more caudally.

Although *P. parnellii and P. quadridens* use comparable heteroharmonic computations, they differ in the cortical organization of neurons according to their best harmonic combination (Figure 2). In *P. parnellii*, delay-tuned neurons are clustered together forming three distinct cortical areas defined as the FM–FM, dorsal fringe and ventral fringe areas. Within the FM–FM and dorsal fringe areas, there is a “harmonic organization” of neurons, i.e., neurons with different best harmonic combinations occur in distinct cortical subdivisions (Suga and O'Neill, 1979). The most ventral subdivision is dominated by neurons tuned to FM1/FM2, the middle subdivision is dominated by neurons tuned to FM1/FM4 and the most dorsal subdivision is dominated by neurons tuned to FM1/FM3 (O'Neill and Suga, 1982) (Figure 2A). In the boundaries between subdivisions, there are “multiple-combination sensitive neurons” that respond maximally when the echo contains combinations of 2nd, 3rd, and 4th biosonar harmonics (Misawa and Suga, 2001). The cortex of *P. quadridens* is different from the cortex of *P. parnellii* in the sense that it is not “harmonically” organized (Hechavarría et al., 2013). In *P. quadridens* only the FM–FM area has been studied and within this area neurons tuned to FM1/FM3 are interspersed with neurons tuned to FM1/FM2 (Figure 2B). One organizational principle shared by the cortices of *P. parnellii* and *P. quadridens* is the “chronotopic” organization of neurons. In these two species, neurons tuned to short echo-delays are located rostrally, while neurons tuned to longer echo-delays are located more caudally (Suga and O'Neill, 1979; O'Neill and Suga, 1982; Schuller et al., 1991; Hagemann et al., 2011; Hechavarría et al., 2013).

Phylogeny studies have shown that *P. parnellii* and *P. quadridens* stem from the most basal and most recent branches in the *Pteronotus* lineage, respectively (Van den Bussche and Weyandt, 2003; Dávalos, 2006). Because of the latter, and the fact both *P. parnellii* and *P. quadridens* possess comparable heteroharmonic neurons, Hechavarría et al. (2013) suggested that the HTCS could be a generalized feature of the genus *Pteronotus* that was preserved during the evolution. The same was suggested for the chronotopic organization of the cortex that is found in both species. On the other hand, harmonically organized chronotopic axes either evolved only in *P. parnellii* or were lost during the evolution of *P. quadridens* (Hechavarría et al., 2013).

Besides *P. parnellii* and *P. quadridens*, heteroharmonic neurons have been found in *R. rouxi* (Schuller et al., 1991). Only neurons tuned to FM1-FM2 were found in this species. Like in *P. parnellii* and *P. quadridens*, in *R. rouxi* there is a clear chronotopic organization of delay tuned neurons. The genus *Rhinolophus* is not closely phylogenetically related to the genus *Pteronotus* (Jones and Teeling, 2006). In fact rhinolophid bats seem to be more phylogenetically related to the megabats than to the remaining microbats (Teeling et al., 2005). The latter suggests that any specialization shared by *Pteronotus* and *Rhinolophus* could be the product of parallel evolution.

Delay-tuning has been studied in other three bat species besides the two *Pteronotus* and *R. rouxi*. In *M. lucifugus, E. fuscus*, and *C. perspicillata* delay tuning seems to be “homoharmonic,” i.e., delay-tuned neurons of these three species respond strongly to pulse-echo combinations of the same harmonic (Sullivan, 1982; Dear et al., 1993; Hagemann et al., 2010). *M. lucifugus* uses a simple FM-pulse for echolocation without prominent harmonics (Griffin, 1962) and therefore it is not surprising that this species uses homoharmonic computations. *E. fuscus* and *C. perspicillata* use biosonar calls that contain at least two harmonics (Thies et al., 1998; Monroy et al., 2011) although call structure can change drastically depending on the behavioral task and the reflective properties of the environment. Yet the delay-tuned of these two species respond strongly to homoharmonic pulse-echo pairs (Dear et al., 1993; Hagemann et al., 2010). Among the homoharmonic species studied so far, only *C. perspicillata* is reported to have a chronotopically organized representation of delay-tuned neurons (Hagemann et al., 2010).

## Brain adaptations for heteroharmonic computations

The mechanisms for the central implementation of delay tuning have been intensively investigated in *P. parnellii* and excellent reviews are available (Wenstrup and Portfors, 2011; Wenstrup et al., 2012). Heteroharmonic delay-tuning is implemented in the auditory midbrain (Wenstrup et al., 2012). Heteroharmonic neurons integrate information from the fundamental biosonar harmonic that provides information about the timing of the pulse and one or several of the upper harmonics in the echo (Portfors and Wenstrup, 1999). Delay-tuned neurons perform as coincidence detectors, i.e., they respond only when there is a temporal coincidence of subthreshold excitations triggered by call and echo. It has been demonstrated that inhibition plays an instrumental role in delaying the response to the call so that it can be aligned in time with the response to the echo. If call-triggered inhibition similarly plays an instrumental role in the implementation of homoharmonic delay tuning is still unknown.

Integrating information from multiple biosonar harmonics is generally accepted as a building block for the implementation of heteroharmonic delay tuning. However, integrating multiple frequency bands (otherwise known as combination sensitivity) is not an exclusive feature of heteroharmonic neurons tuned to echo-delay. For example, combination sensitive responses have been found in mice, birds, monkeys and homoharmonic bat species, among others (Margoliash and Fortune, 1992; Dear et al., 1993; Rauschecker et al., 1995; Hernández et al., 2005; Portfors and Felix, 2005; Felix and Portfors, 2007; Hagemann et al., 2010). The currently available data suggests that combination sensitivity is a generalized principle of the mammalian auditory system that was further used by heteroharmonic bats for the implementation of a specialized strategy for target-distance computation.

## Call design and target range

The examination of call design could provide a better understanding of the evolution of the HtHCS for target-range computation in bats and specifically in the family Mormoopidae. Bats use a highly diverse repertoire of call designs. Biosonar call diversity is observed both across (e.g., Schnitzler et al., 2003) and within species (e.g., Mora et al., 2011). One approach for categorizing bat calls distinguishes short FM from long CF calls. Typically, bats that broadcast pure-FM calls listen for echoes before emitting the next call to avoid temporal overlapping of call and echo. This calling strategy maintains a low duty cycle (LDC) (i.e., the proportion of time occupied by biosonar calls during an echolocation sequence is <25%). On the other hand, bats that use long CF echolocation calls separate call and echo in the frequency domain (because of the Doppler shifted echo). CF-bats are able to broadcast calls and receive echoes at the same time and therefore they can use HDC echolocation, with duty cycle values above 25%. Call design is tightly linked to duty cycle. Most echolocating bats use LDC echolocation (Fenton et al., 2012). HDC echolocation is a feature of only a few bats species (i.e., species from the families Rhinolophidae and Hipposideridae, and *P. parnellii* from Mormoopidae). Only the family Mormoopidae includes both LDC and HDC species. Although useful as a first approach, classifying bats into FM-LDC and CF-HDC according to their calling strategy is not fine-grained enough to explore the evolution of target-range computation in mormoopids.

**Figure 3:**
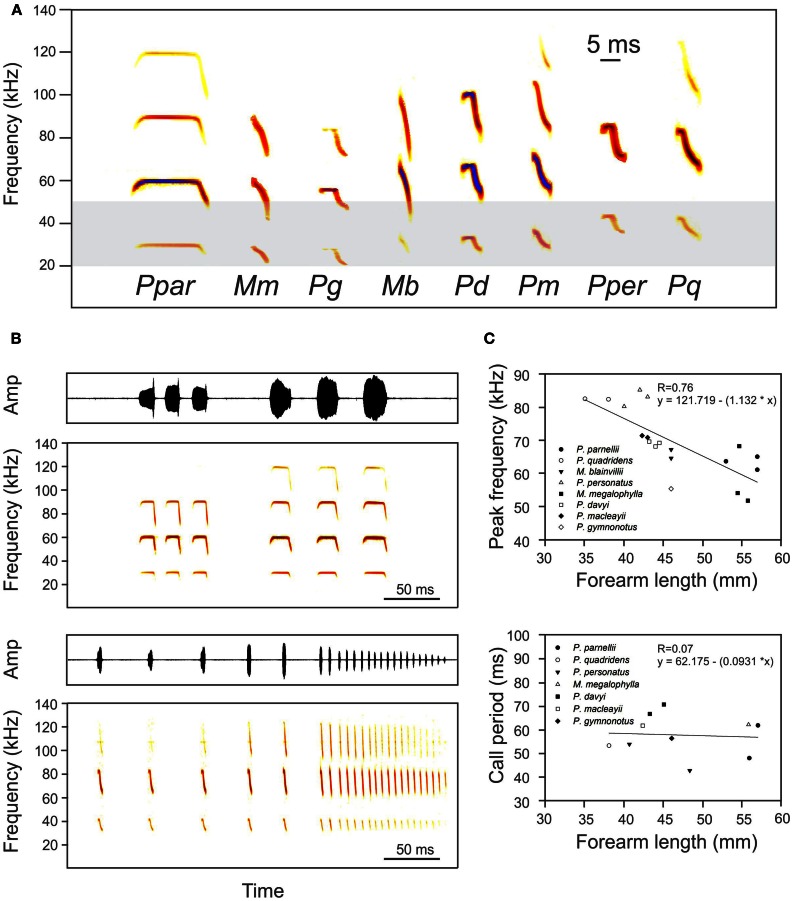
**(A)** Spectrograms of typical search calls of the eight bat species of the family Mormoopidae (Ppar: *Pteronotus parnellii*, Mm, *Mormoops megalophylla*; Pg, *Pteronotus gymnonotus*; Mb, *Mormoops blainvillei*; Pd, *Pteronotus davyi*; Pm, *Pteronotus macleayii*; Pper, *Pteronotus personatus*; Pq, *Pteronotus quadridens*). The light-gray area represents the frequency range of best audition in eared moths, after Fullard (1988). **(B)** Typical echolocation sequence (oscillogram and spectrogram) emitted by *P. parnellii* (up) and *P. quadridens* (down) during foraging. Note that call harmonics never overlap. **(C)** Relationships between peak frequency (up) and call period (down) and forearm length for the eight species of mormoopid bats. Lower frequency calls are emitted by larger bats. Call period is independent of body size and signal design. Data was taken from: Silva-Taboada (1979), Herd (1983), Adams (1989), Rodríguez-Durán and Kunz (1992), Rezsutek and Cameron (1993), Lancaster and Kalko (1996), O'Farrell and Miller (1997), Ibañez et al. (1999), Ibañez et al. (2000), Macías et al. (2006), Smotherman and Guillen-Servent (2008), MacSwiney et al. (2008), de la Torre and Medellin (2010), and Mancina et al. (2012).

Call design is polymorphic within the family Mormoopidae. FM calls are emitted by the two species of the genus *Mormoops*, long CF-FM calls are emitted by *P. parnellii* and sCF-FM and FM-sCF calls are emitted by the other five species of the genus *Pteronotus*: *P. personatus*, *P. davyi*, *P. gymnonotus*, *P. macleayi*, and *P. quadridens* (Fenton, 1994; O'Farrell and Miller, 1997; Ibañez et al., 1999, 2000; Kössl et al., 1999; Macías and Mora, 2003; Macías et al., 2006; Smotherman and Guillen-Servent, 2008; Mora and Macías, 2011) (Figure 3A). For ranging, the following parameters of signal design are expected to be of special importance: (1) the number of harmonics, (2) the frequency overlap of harmonics, (3) the bandwidth of the FM component, (4) the duration and curvature of the FM component, and (5) the frequency range and intensity of each FM-component.

Obviously, a heteroharmonic mechanism for target-range computation can only operate on call-echo pairs with at least two harmonics. The two mormoopids in which the HtHCS has been reported [i.e., (*P. parnellii* and *P. quadridens*)] broadcast calls with three or more harmonics, as do the remaining *Pteronotus* and *Mormoops* species (Figure 3A). Also, the echolocation calls of *R. rouxi* (the third species in which HtHCS has been described) contain two harmonics (Neuweiler et al., 1987). *E. fuscus* and *M. lucifugus* (two homoharmonic species) use echolocation calls with one or two harmonics (Moss et al., 1997; Surlykke and Moss, 2000). Besides Mormoopidae, other families that use multiharmonic echolocation calls (i.e., three or more harmonics) are Megadermatidae, Nycteridae, and Phyllostomidae (Jones and Teeling, 2006; Jones and Holderied, 2007). It is known that at least one phyllostomid species (*Carollia perspicillata*) uses the HmHCS (Hagemann et al., 2010). Therefore, broadcasting multiple harmonics does not seem to be sufficient for using the heteroharmonic target-distance computations.

In the three species known to use HtHCS, there is no overlapping between the harmonics of the FM-component of the calls (Figures 3A,B). Therefore the ability of calling (and hearing) in spectrally independent bands could be a prerequisite for using HtHCS. Supporting this idea is the fact that *C. perspicillata* (which uses HmHCS) uses multiharmonic calls with harmonic overlapping (Thies et al., 1998). The echolocation calls from all mormoopid species show non-overlapping harmonics. Non overlapping harmonics are also observed in the biosonar calls of rhinolophids and hipposiderids.

To be able to keep harmonics fully separated in the frequency domain, bats need to limit the bandwidth of their FM calls or components. Increasing bandwidth is appropriate to develop a detailed acoustic snapshot of the surrounding and to separate prey from background clutter (Simmons and Stein, 1980; Siemers and Schnitzler, 2004). It is known that most bat species are capable of adjusting call bandwidth according to echolocation task. In Rhinopomatidae (e.g., Habersetzer, 1981), Vespertilionidae (e.g., Kalko and Schnitzler, 1993), Molossidae (e.g., Mora et al., 2011), Emballonuridae (e.g., Kalko, 1995) and Phyllostomidae (e.g., Mora and Macías, 2007) for example, several species can adjust the bandwidth of their calls to broadcast from quasi-constant frequency calls (BW < 4 kHz) to wideband FM calls (BW > 15 kHz) by adjusting the frequency band of their FM components. Mormoopids are different; they keep the bandwidth of their calls remarkably constant (Figure 3B) (but see *Mormoops*: Macías et al., 2006; Smotherman and Guillen-Servent, 2008), thus avoiding harmonic overlap (Macías and Mora, 2003; Macías et al., 2006; Mora and Macías, 2011).

Not only the bandwidth of the FM-sweep but also its duration and curvature may affect the estimation of target-range and influence the performance of the computation process. Both from the “distance of focus” theory (Boonman et al., 2003; Holderied et al., 2006) and from behavioral (Simmons, 1973) and neurophysiological data (Jen and Wu, 2008), there is evidence showing that short calls and echoes are more appropriate for an accurate estimation of short target-distances, which might decrease collision risks and increase the probability of a successful capture. For a bat flying faster than 3.4 m/s (1% of the speed of sound), Doppler effects will lead to a distortion of the perceived range due to compression of echo delay time and elevation of echo frequency. However, the accuracy of short target-distance estimation increases if short hyperbolic FM calls are used, and also if strong harmonics are added (Boonman et al., 2003; Simmons et al., 2004). To the best of our knowledge mormoopid calls have not yet been used to investigate how signal design could affect the measurement of echo-delay at different flight speeds as it has been done in other species (Simmons, 1973; Altes, 1980; Boonman et al., 2003). Nonetheless the visual inspection of mormoopid calls suggests a call structure suited to minimize errors in measuring distance caused by Doppler Effect, mainly if the FM-component is taken into account (Figure 3B). The CF-component of variable length in the mormoopid calls will effectively widen the envelope of the cross-correlation function, causing Doppler tolerance to decrease (Simmons, 1973). However, in a filter bank model Doppler tolerance will not decrease dramatically by adding a CF-component to the wideband FM-component, since this affects only a portion of the receiver channels (Boonman et al., 2003). In other words, the CF-component, thought to be used in the estimation of relative velocity and the recognition of fluttering insects (Schuller, 1984; Suga, 1990; review: Schnitzler and Denzinger, 2011), and the FM-component, used to measure target-distance (Simmons, 1973; Saillant et al., 1993), must be analyzed independently.

An additional ranging error is expected while flying since bats approach the target as the reflected echoes travel to the bat ears. Since this error causes an underestimation of target range while the Doppler-related error causes an overestimation of range, they cancel each other at a certain target distance (defined as the distance of focus; Boonman et al., 2003). By adjusting the design of the FM-sweep during flight in a range dependent way, bats can avoid these sources of error so that nearby objects are localized accurately, a behavior termed focusing (Boonman et al., 2003; Holderied et al., 2006). Future studies will show if mormoopids employ acoustic focusing and if they are able to adapt the duration, bandwidth, and curvature of FM biosonar elements to cancel out flight-speed-related ranging errors as a function of target-distance. If that is the case, delay-tuned neurons might show sharper delay tuning curves the shorter the “distance of focus” of the FM call-echo pairs used as acoustic stimuli in the neurophysiology experiment, improving the positive correlation between the best delay and the width of the delay tuning curves (see Figure 1) already observed in the auditory cortex (Suga and Horikawa, 1986; Hagemann et al., 2010; Hechavarría et al., 2013). Whether heteroharmonic bats correlate the returning echo with the actual outgoing call, or whether “hard-wired” replicas of the bat's characteristic signals are contained in the auditory system remains a very interesting question to be solved.

There is evidence that call design in mormoopids is linked to the HtHCS. Duty cycle, however, is not. In other words, call design may need to fulfill certain requirements for the bat to operate the HtHCS, but the HtHCS can operate both in LDC and HDC echolocation. For more than three decades (O'Neill and Suga, 1979) the heteroharmonic target-range computation was known only for two CF-HDC bats. However, the sCF-FM-LDC *P. quadridens* also computes call-echo delay heteroharmonically (Hechavarría et al., 2013). The relatively high proportion of the “on time” of the call is achieved in HDC rhinolophids and hipposiderids by increasing the duration of the call relative to the call period (i.e., the time between the onset of successive calls) (Fenton et al., 2012). However, call period in most insectivorous bat species studied to date, is determined by the species wingbeat period (Speakman and Racey, 1991). If the same applies to the CF-HDC mormoopid *P. parnellii* then call period would remain at values equivalent to those in the other species of the family, as it is shown in Figure 3C. What is of relevance for target-range computation is the time interval between the emissions of two consecutive FM-components. Therefore, it is not surprising that the general rules that govern the temporal parameters of the calling strategy in FM-HmHCS bats also govern those of CF-HtHCS bats if only the FM-components are taken into account, i.e., as bats get closer to targets, they shorten the call's duration (or that of the FM-component) and the interval between calls (or between FM-components) thus increasing ranging performance (Boonman et al., 2003) and the accuracy in the estimation of the target's angular position (Suga, 1990). In consequence, once CF-HDC bats detect, lock and start tracking fluttering insects, the CF-component will shorten principally to accommodate the temporal changes of the FM-component that will rule the distance-to-target dependent temporal adaptations of the bat calling behavior.

In conclusion, it seems likely that any bat making use of the HtHCS will broadcast FM calls (or calls with FM components) with two or more harmonics without frequency overlap. In addition, it is of advantage if the duration and curvature of the FM components are adjusted for acoustic focusing as a function of distance to target, in correlation with neuronal adaptations for the processing of call design as a complement of the target-range computation strategy. Also it could be predicted that bats that use HtHCS are capable of a precise control of call frequency and intensity. The latter will be explored in the following section.

## Frequency and intensity of call and echo

Echolocating bats dynamically change the acoustic parameters of calls (i.e., frequency, intensity, temporal parameters) to cope with their environment and perceptual task. A closer view at the common principles used by HtHCS bats to exploit frequency and intensity of calls- and echoes- may help to assess the evolution of their target-range computation strategy. This section focuses on the analysis of frequency and intensity because both parameters are closely related in the heteroharmonic target-range computation strategy. The frequency spectrum of each call is determined by the amount of energy or sound intensity distributed between harmonics, and frequency and intensity are the two main parameters used to characterize the receptive field of delay-tuned neurons (see section Delay Tuning in Auditory Neurons of Different Bat Species).

The most obvious difference between HtHCS and HmHCS bats is in the frequency content of interest for assessing the timing of calls and echoes. Species that compute target-range homoharmonically broadcast and listen in the same frequency band since relevant wavelengths in calls and echoes are the same (Simmons, 2012). In contrast, heteroharmonic bats always pay attention to the fundamental harmonic in the call but to the higher order harmonics in the echoes. All HtHCS bats focus energy in higher harmonics but assign very little (as little as 1% of the total energy) to the fundamental harmonic (Figure 3A). The bat will still hear the faint fundamental harmonic of its call due to the small distance between mouth and ear, and the relatively weak attenuation of low frequencies (Lawrence and Simmons, 1982). However, conspecifics will mainly hear the higher harmonics. Attenuating the fundamental harmonic in HtHCS bats could minimize call-echo interference in bat colonies with hundreds or thousands of individuals since FM-components of higher harmonics by themselves cannot excite FM–FM neurons (Suga, 1990). The high frequency FM-components of the echoes will only elicit auditory responses in delay-tuned neurons if the calling bat have previously emitted and listened to its own fundamental harmonic. In the Caribbean islands, mormoopid bats are dominant in cave ecosystems where they enjoy the advantages of living in large colonies (Silva-Taboada, 1979; Goerlitz et al., 2012; Lima and O'Keefe, 2013). However, it is worth mentioning that the largest bat colonies known to mankind are of presumed homoharmonic species i.e., *Tadarida brasiliensis* (Betke et al., 2008; Hristov et al., 2010). Future research is needed to unveil how HmHCS bats deal with target-range computation in environments with so much overlapping frequency interference.

Rather fixed frequency-limits of FM-components also distinguish HtHCS from HmHCS bats. Mormoopids, but also rhinolophids and hipposiderids, keep the maximum frequency of their FM-components at the value of their CF-components (Figures 3A,B). In mormoopids flying in open spaces, even the minimal frequency of each FM-component seems to be restricted by the addition of a lower sCF-component to the call (O'Farrell and Miller, 1997; Mora and Macías, 2011). In contrast, frequency limits of individual harmonics are less fixed in HmHCS bats that vary either the maximal and/or the minimal frequencies of the emitted calls to adjust bandwidth (Kalko and Schnitzler, 1993; Surlykke and Moss, 2000; Mora et al., 2005).

The most widely used hypothesis to explain dominant call frequencies in bats is the *allometry hypothesis* (Jones, 1996, 1999). Due to the physics of sound, the structures associated with sound production generate lower-frequency sounds as size increases (Pye, 1979), and therefore it is predicted that larger bats emit at lower frequencies. In Mormoopidae, call frequency scales negatively with body size (i.e., forearm length) (Figure 3C). Since the *allometry hypothesis* explains call frequencies in several other bat families including the presumed HtHCS Rhinolophidae and Hipposideridae (Heller and Helversen, 1989; Jones, 1999), this hypothesis is not of much value to explore the evolution of the heteroharmonic strategy.

The *allotonic frequency hypothesis* suggests that relatively high or low echolocation frequencies are the result of selection to become less audible to eared insects, especially moths (Fullard, 1987). Tympanate moths have maximum hearing-sensitivity between 20 and 50 kHz (Fullard, 1988) which coincides with the frequency range echolocation calls of most bat species (Fenton et al., 1998). The fundamental biosonar harmonic of each mormoopid species contains frequencies syntonic (i.e., between 20 and 50 kHz) with moth hearing, but due to its relatively low intensity it may be barely detectable by the prey, thus offering a good example of harmonic-dependent stealth echolocation (Goerlitz et al., 2010). If multiharmonic echolocation evolved in mormoopids to allow these bats to exploit the soft nutritious moths as a food resource, then it would ideally combine faint first syntonic harmonics with loud high-frequency allotonic harmonics. Such a call would be optimally designed to overcome prey hearing (Figure 3A). In fact, several studies have shown that moth constitutes a major prey item in the diet of many Caribbean mormoopids (Silva-Taboada, 1979; Rolfe and Kurta, 2012). Therefore, hearing-mediated detection of bats by moths could have operated as an important evolutionary force for the acquisition of the heteroharmonic target-range computation strategy in Neotropical mormoopids.

The frequencies used by HtHCS mormoopids may be also explained by the *prey detection hypothesis* (Houston et al., 2004) which relates the strength of an echo with the wavelength of the call and the dimensions of the prey. The non-overlapping harmonics of the call theoretically allow mormoopids to exploit a broad range of prey sizes. For example, it is generally accepted that insects generate relatively strong echoes from biosonar wavelengths that match the dimensions of their prominent scattering points (i.e., head and wings). If the latter is true, *P. quadridens* could target a variety of insects with size differences of about 3 mm according to echoes from the minimum frequencies of the second harmonic (61.22 kHz, wavelength 5.5 mm) and from the maximum frequencies of the third harmonic (124.00 kHz, wavelength 2.7 mm). Distinction of insect size will be favored by individual auditory neurons responding to either the echoes from the second or the third harmonics (see Figures 1A,B). Smaller preys could be detected by adding a fourth harmonic to the call, which will significantly increase strength of echoes generated in smaller insects (Houston et al., 2004). Frequency-dependent atmospheric attenuation, however, would be a serious limitation in the use of high-order harmonics, but negligible at short range where it has been found that mormoopid bats incorporate a third and even fourth harmonic to their vocalizations (Macías and Mora, 2003; Mora and Macías, 2011). We argue that bats using HtHCS get a bonus in the categorization of insect size by focusing acoustic energy in discrete harmonic bands which in addition safes energy.

Two other hypotheses have been used to explain the frequency composition of biosonar calls: the *foraging habitat hypothesis* (Jones and Barlow, 2004) and the *acoustic communication hypothesis* or *acoustic resource partitioning hypothesis* (Duellman and Pyles, 1983; Heller and Helversen, 1989). According to the foraging habitat hypothesis, bats species that forage in more-cluttered habitats should use calls of higher frequencies than species foraging in less cluttered/more-open habitats (Stoffberg et al., 2011). Due to the multiharmonic structure of mormoopid calls this hypothesis is of limited value for explaining the emission of high frequencies in relation to clutter; i.e., high frequency demands are solved in HtHCS species by adding more harmonics. However, it is important to note that *P. parnellii* (a species that forages in highly-cluttered environments), uses one of the lowest frequencies within the genus *Pteronotus* (Figure 3A). The *acoustic communication hypothesis* predicts that different frequencies could evolve under selection pressures imposed during social interactions (Heller and Helversen, 1989; Thabah et al., 2006). In our opinion, this hypothesis does not add new insights to the evolution of the target-range mechanism in Mormoopidae.

Doppler shift compensation is not linked to the HtHCS. DSC involves lowering the frequency of the next echolocation call to compensate for the flight-induced increase in the frequency of echoes from a previous emission (Schnitzler and Denzinger, 2011). By compensating for Doppler effects, bats ensure that the CF-component of the echoes remains within the range of frequencies to which their auditory system is most sensitive, i.e., the “auditory fovea” (Neuweiler, 1990, 2003). The frequency value for which the auditory fovea shows its highest sensitivity is defined as the resting frequency (Suga and Jen, 1976; Smotherman and Guillen-Servent, 2008). The CF-FM *P. parnellii* compensates for Doppler shifted echoes (Henson et al., 1980) but the sCF-FM *P. quadridens* does not (Mora and Macías, 2011). However, these two species measure target-distance using the HtHCS. It is tempting to predict that DSC will have an influence on the target-range computation of mormoopids. For example, in neurophysiological experiments with species showing DSC such as *P. parnellii* and *P. personatus*, the best responses of delay-tuned neurons might occur when the bat is presented with call-echo combinations in which the call's fundamental harmonic (FM1) is lowered in frequency and the echo's higher harmonics (FMx) is set at the species resting frequency. In contrast, the non-compensating smaller mormoopids should show best responses of delay-tuned neurons for combinations of the call's resting FM1 and the echoes' shifted FMx.

Changes in the amplitude of call and echo are also relevant for the target-range computation mechanism. During flight, both HmHCS and HtHCS echolocating bats decrease the intensity of their emitted pulses when approaching a prey item or an obstacle (Kobler et al., 1985; Boonman and Jones, 2002; Hiryu et al., 2007, 2008). Call intensity is adjusted in relation to the distance to target while maintaining echo intensity within an optimal sensitivity range. This intensity compensation will surely affect the shape of the response areas of delay-tuned neurons (DRAs, see section Delay Tuning in Auditory Neurons of Different Bat Species), that so far have been obtained by keeping constant the level of the call while changing the level of the echo (Suga, 1990; Hagemann et al., 2010; Kössl et al., 2012; Hechavarría et al., 2013). It is expected that in both HtHCS and HmHCS bats, lower call intensities will shift best call-echo delays to shorter values. Previous results from neurophysiology experiments in the HmHCS bat *E. fuscus* are in agreement with this prediction (Jen and Wu, 2008).

Intensity compensation has been mainly analyzed for whole calls and echoes, regardless of the species that is studied (Hiryu et al., 2007, 2008; Surlykke and Kalko, 2008). However, in bats using the HtHCS, the intensity compensation and its effect on target-range computation, need to be analyzed on a harmonic level. The changes of call/echo intensity in the fundamental harmonic may not be the same in the second or higher harmonics. Combining the acoustic/neuronal rules that seem to describe the HtHCS with those of intensity compensation, two main predictions arise: (1) that the intensity of the fundamental harmonic of the calls will remain stable while the intensity of the echo will increase as bats approach targets; and (2) that the intensity of the call's second or higher harmonics will decrease while the amplitude of the correspondent echoes will remain stable as bats approach targets.

## Heteroharmonic echolocation in the phylogeny of mormoopidae

**Figure 4:**
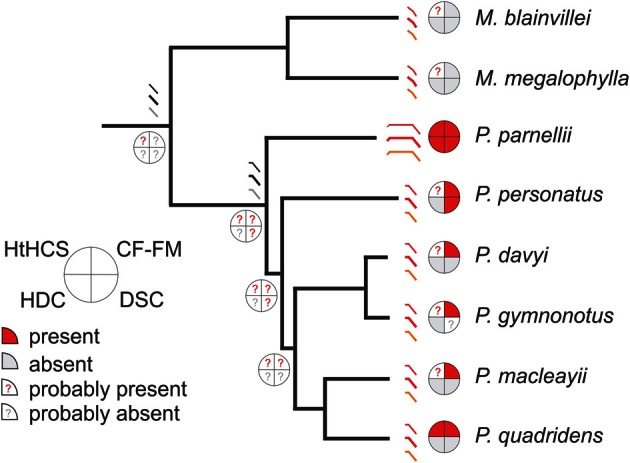
**Echolocation traits of mormoopid bats mapped onto the molecular phylogenetic tree of the family, after Van den Bussche and Weyandt (2003) and Dávalos (2006).** Schematics of echolocation calls from extant species (in red) and suggested common ancestors (in gray) have been represented. The heteroharmonic computation strategy is expected to be found in each species of the family, in contrast to the other three traits that characterize only some of the extant species. The evolutionary position of *P. parnellii* points to the long-CF calls and HDC as evolutionary singularities.

To summarize the ideas discussed in the present review four important echolocation traits for mormoopid bats were mapped on a phylogenetic tree (Figure 4) (adapted from molecular data from Van den Bussche and Weyandt, 2003 and Dávalos, 2006). Mormoopid echolocation is characterized by quite diverse call designs and biosonar strategies that outside Mormoopidae distinguish different bat families. This diversity offers the opportunity to revise each species echolocation with the intention of tracking the ancestral condition and the evolutionary paths of each sonar trait. In some cases where there is not sufficient supporting data available, the two categories, “probably present” or “probably absent,” are used to be able to speculate on phylogenetic trends. Future research is needed to fill the gaps in knowledge and to evaluate the present speculations.

The first look to the echolocation in Mormoopidae (Figure 4) confirms non-overlapping harmonics in the calls of each species of *Pteronotus*. Non-overlapping harmonics have been identified in this review (see sections Brain Adaptations for Heteroharmonic Computations and Call Design and Target Range) as a promising feature that would support the HtHCS for target-range computation. In addition, both the most basal and one of the most recently evolved species of *Pteronotus* computes call-echo delay heteroharmonically (Suga, 1990; Hechavarría et al., 2013). As mentioned in the preceding text (section Introduction), it is likely that every other *Pteronotus* species will also use the HtHCS for ranging. In addition, we speculate that both *Mormoops* species will also compute target-range heteroharmonically. Not only are the echolocation calls of *Mormoops* of a multiharmonic structure but also they contain a prominent second harmonic and a faint first harmonic (O'Farrell and Miller, 1997; Macías et al., 2006), features that as dicussed here could be related to the HtHCS. Indirect support for a HtHCS comes from the observation that *Mormoops* lives in large colonies and is a specialized moth predator (Silva-Taboada, 1979; Goerlitz et al., 2012; Rolfe and Kurta, 2012), behaviors that might profit from using heteroharmonic computations (Suga, 1990). Arguing against the possibility of finding a heteroharmonic strategy in *Mormoops* is the fact that in this genus, the echolocation calls broadcasted while approaching a target show some degree of frequency overlapping (Macías et al., 2006; Smotherman and Guillen-Servent, 2008). Frequency overlapping is not observed in the echolocation calls of *Pteronotus* species (Macías and Mora, 2003; Macías et al., 2006; Smotherman and Guillen-Servent, 2008; Mora and Macías, 2011). However, if the HtHCS is finally demonstrated in *Mormoops*, it will support the theory that the common ancestor of *Pteronotus* and *Mormoops* already featured this echolocation trait.

The other three mormoopid echolocation traits, i.e., CF-FM calls, DSC and HDC are restricted to some mormoopid species (Figure 4). CF-FM calls (regardless of the duration of the CF component) are typical of *Pteronotus* and not of *Mormoops*, but using a long CF-component is a unique characteristic of *P. parnellii*. We therefore propose that the common ancestor of *Pteronotus* featured CF-FM calls. In this context, the long CF calls of the mustached bat, that allowed the species to echolocate at HDCs, are better explained as an evolutionary singularity probably produced by genetic change that introduced specialized modifications in cochlear development leading to an exceptionally sharp tuning to the CF call component [see discussion in Vater (1999); Kössl et al. (1999)]. DSC could have also characterized the *Pteronotus* ancestor since both *P. parnellii* and *P. personatus* compensate for flight-induced frequency shifts (Smotherman and Guillen-Servent, 2008; review: Schnitzler and Denzinger, 2011). DSC in *P. parnellii* has been interpreted as instrumental to assure the processing of CF echoes carrying information about fluttering insects by an exceptionally sharply tuned auditory fovea (reviews: Neuweiler, 1990, 2003). There are no previous studies on the auditory system of *P. personatus*, but at least a disproportionate representation of neurons processing the resting sCF-component frequency and an enhanced sensitivity to this frequency range is to be expected. If it is assumed that the common ancestor of the genus *Pteronotus* already possessed DSC, it would be quite challenging to explain the loss of DSC in the most recent *Pteronotus* species. The most parsimonious hypothesis would be that the foraging strategy adopted by the smaller *Pteronotus* relies upon a more broadly tuned auditory system (Kössl et al., 1999) and like most FM bats, they can tolerate modest Doppler effects (Boonman et al., 2003). A detailed analysis of the possible evolutionary scenario for the acquisition of DSC is beyond the scope of this work, but excellent reviews on this echolocation attribute are available (Schnitzler and Denzinger, 2011; Fenton et al., 2012).

Phyllostomidae is a sister family of Mormoopidae (Teeling et al., 2003; Eick et al., 2005). Because mormoopids are heteroharmonic (Hechavarría et al., 2013) and phyllostomids homoharmonic (Hagemann et al., 2010, 2011), it is difficult to infer the ranging strategy of the common ancestor of the two families. No indications for homoharmonic echolocation are apparent within the family Mormoopidea. Therefore if the ancestor of Mormoopidae was homoharmonic, this strategy was completely replaced by the heteroharmonic strategy during the evolution of the family. On the other hand, if the ancestor of Mormoopidae used HtHCS, some evidence could still be found within the many species of Phyllostomidae. Since *C. perspicillata* uses the HmHCS, and this species is relatively recent in the phylogeny of phyllostomids (Rojas et al., 2011), one should look into more ancient taxa to try to find any indication of HtHCS. *Macrotus*, the most basal genus of Phyllostomidae, could by the right taxon to find out whether the HtHCS was lost before the first phyllostomids appeared or during their evolutionary history. The two extant species of *Macrotus* (*Macrotus californicus* and *Macrotus waterhousii*) are gleaning bats that emit multiharmonic calls with faint fundamental harmonics, but showing frequency overlap (Murray et al., 2009).

Outside the New World, the same features characterizing the echolocation of Mormoopidae are found in species of the families Rhinolophidae and Hipposideridae. CF-FM, DSC and HDC in those bat families and in Mormoopidae are frequently taken as good examples of convergent evolution to emphasize how perceptual challenges imposed by the environment can override phylogenetic constraints (Jones and Teeling, 2006; Jones and Holderied, 2007). Rhinolophid bats make use of the HtHCS for ranging, long CF-FM calls, high duty-cycle and Doppler shift compensation (review: Schnitzler and Denzinger, 2011). Hipposiderids show similar echolocation traits but with shorter CF calls, lower duty cycles and a less advanced DSC. Their calls show the same signal structure suggested here to be necessary to perform the HtHCS.

## Conclusions and future directions

This review presents the hypothesis that the HtHCS for target-range estimation assisted the ancestors of mormoopid bats in categorizing target size, hunting for eared prey and inhabiting caves in large numbers. We suggest that the implementation of the HtHCS evolved in parallel to the ability of using CF calls, HDC echolocation, and DSC behavior. The detailed analysis of echolocation signal design and its task-dependent adaptations in acoustic parameters, on top of recent gene-based phylogenies obtained for the species in the family Mormoopidae, allow the identification of common principles in the evolution of target-range computation in mormoopids and other heteroharmonic bats. The following predictions might help to define some of the evolutionary building blocks for this echolocation strategy.

1. *Each species of the genera Pteronotus and Mormoops is predicted to perform HtHCS*. This is supported by the findings that within Mormoopidae, the most ancient and the most recent lineages show HtHCS and within Moormopidae call designs are similar across species.
2. *Mormoopids should be able of dynamic harmonic hopping, i.e., individuals can shift energy between the high order harmonics*. In theory, the HtHCS supports harmonic hopping to minimize high interference (i.e., from conspecifics) or to aid in the discrimination of different target sizes.
3. *If the ability of HtHCS computation characterized the ancestors of Noctilionoidea it may have prevailed at least in descendent species with limited frequency overlap between harmonics*. The genera *Noctilio* and *Macrotus* are appropriate candidates to test this hypothesis.
4. *Intensity compensation is harmonic-dependent in mormoopids and other bats with HtHCS*. Calls and echoes represent different harmonic interests for the heteroharmonic echolocator and therefore the rules describing the dynamic adjustment of call/echo intensity will distinguish one harmonic from the other.
5. *If the echolocation calls of mormoopids evolved to hunt eared prey, they will be relatively inaudible to moths if compared to calls from HtHCS bats of comparable size*.

### Conflict of interest statement

The authors declare that the research was conducted in the absence of any commercial or financial relationships that could be construed as a potential conflict of interest.

## Abbreviations

HtHCS: heteroharmonic computation strategy
HmHCS: homoharmonic computation strategy
HDC: high duty-cycle
LDC: low duty-cycle
CF: constant frequency
FM: frequency modulation
DSC: Doppler shift compensation.
